# Sex Differences in Clinical Presentation and Outcomes among Patients with Complement-Gene-Variant-Mediated Thrombotic Microangiopathy

**DOI:** 10.3390/jcm9040964

**Published:** 2020-03-31

**Authors:** Christof Aigner, Martina Gaggl, Renate Kain, Zoltán Prohászka, Nóra Garam, Dorottya Csuka, Raute Sunder-Plassmann, Leah Charlotte Piggott, Natalja Haninger-Vacariu, Alice Schmidt, Gere Sunder-Plassmann

**Affiliations:** 1 Division of Nephrology and Dialysis, Department of Medicine III, Medical University Vienna, 1090 Vienna, Austria; martina.gaggl@meduniwien.ac.at (M.G.); lcpiggott@gmail.com (L.C.P.); natalja.haninger@meduniwien.ac.at (N.H.-V.); alice.schmidt@meduniwien.ac.at (A.S.); gere.sunder-plassmann@meduniwien.ac.at (G.S.-P.); 2 Department of Pathology, Medical University Vienna, 1090 Vienna, Austria; renate.kain@meduniwien.ac.at; 3 Research Laboratory, 3rd Department of Internal Medicine, and MTA-SE Research Group of Immunology and Hematology, Hungarian Academy of Sciences and Semmelweis University, 1094 Budapest, Hungary; prohaszka.zoltan@med.semmelweis-univ.hu (Z.P.); norigaram@gmail.com (N.G.); csuka.dorottya@med.semmelweis-univ.hu (D.C.); 4 Genetics Laboratory, Department of Laboratory Medicine, Medical University Vienna, 1090 Vienna, Austria; raute.sunder-plassmann@meduniwien.ac.at

**Keywords:** genetic renal disease, clinical nephrology, hemolytic uremic syndrome, thrombotic microangiopathy, orphan disease, sex, gender

## Abstract

Sex differences among patients with complement-gene-variant-mediated thrombotic microangiopathy (cTMA) are not well established. We examined demographic and clinical data from female and male patients with a history of cTMA enrolled in the Vienna thrombotic microangiopathy (TMA) cohort. Follow-up was three years after first presentation with cTMA. In this single-center study, we identified 51 patients with a first manifestation of cTMA between 1981 and 2019; 63% were female (*p* = 0.09). The median age at diagnosis did not differ between females and males. There was also no disparity between the sexes with regard to renal function or the need for renal replacement therapy at presentation. Furthermore, we observed similar use of plasma or eculizumab therapy and a comparable evolution of renal function of female and male patients. More females showed risk haplotypes of complement factor H (*CFH*) and *CD46* (97% vs. 68%, *p* = 0.01), but there was no difference in the prevalence of rare pathogenic variants in complement-associated genes with regard to sex. In conclusion, the majority of cTMA patients enrolled in the Vienna TMA cohort were female. Clinical presentation and renal function did not differ between the sexes, but females more frequently presented with cTMA risk haplotypes.

## 1. Introduction

Complement-gene-variant-mediated thrombotic microangiopathy (cTMA or atypical hemolytic uremic syndrome, aHUS) is a rare hereditary and devastating disease, resulting in kidney failure and premature death [1,2,3]. It is characterized by microangiopathic hemolytic anemia, thrombocytopenia, and organ damage due to an alternative complement pathway overactivation within the vascular endothelium [4]. Treatment with plasma exchange and, more recently, eculizumab, an anticomplement factor 5 antibody, has improved renal outcome and patient survival tremendously [5,6].

Among adult patients, the incidence of cTMA is estimated to be less than one case per million [2] and could be higher in females as compared to males, because pregnancy is an important trigger for the development of cTMA [7,8,9,10]. However, sex differences in the presentation and outcome of cTMA are not well defined. In this context, a recent analysis from the International aHUS Registry found that only 9% of females had a pregnancy before disease manifestation [11]. Since multicentric registry data are frequently incomplete, analysis of well-defined single-center cohorts may be more reliable in this regard.

The objective of this study was to examine sex differences in the presentation and outcomes of cTMA patients enrolled in the Vienna thrombotic microangiopathy (TMA) cohort, which is a large single-center population comprising patients with all kinds of TMA, such as cTMA, thrombotic thrombocytopenic purpura, or secondary TMA, of various origin.

## 2. Methods

### 2.1. Study Population

The Vienna TMA cohort was established in 2014 at the Division of Nephrology and Dialysis, Department of Medicine III, Medical University of Vienna [12,13,14]. We continuously include, among other types of TMA, patients with a diagnosis of cTMA [1].

For this analysis, we included all patients with a diagnosis of cTMA, as defined by Fakhouri et al., that presented to our center between 1981 and September 2019 [2]. Secondary TMA was diagnosed if any risk factors were identified, as described in Aigner et al. [1]. Demographic, clinical, and genetic data were retrieved from the electronic and paper-based health care records of our institution. The institutional review board at the Medical University of Vienna approved the study (identifier: 1368/2014). Patients that were prospectively included in the study gave written consent.

### 2.2. Laboratory Methods

The standard laboratory work-up was carried out at the Department of Laboratory Medicine, Medical University of Vienna. Complement-specific laboratory work-up (levels of complement factors, CFH autoantibodies, etc.) was performed at the laboratory of the 3rd Department of Internal Medicine, Semmelweis University in Budapest, using previously published methods [15]. We used genetic sequencing and multiplex ligation-dependent probe amplification to search for genetic variants in the following genes: *CFH*, *CFI*, *CD46*, *C3*, *CFB*, *THBD, CFHR1, CFHR3-5,* and *DKGE*. Genetic analysis was performed at the Department of Laboratory Medicine at the Medical University of Vienna and the research laboratory of the 3rd Department of Internal Medicine, Semmelweis University in Budapest, as previously described [12].

### 2.3. Classification of Sequence Variants

Rare genetic variants were categorized for their pathogenicity using published experimental evidence, reference allele frequencies, and in silico prediction, in accordance with genetic variant guidelines [16]. We also used the “Database of Complement Gene Variants” for classification as pathogenic, likely pathogenic, uncertain significance, likely benign, or benign. Common genetic variants in aHUS-related genes were defined according to Osborne et al., who identified 14 variants with an allele frequency of >1% in at least one of three reference datasets—1000GP, EVS, and ExAC [9]. We used the prediction tools PolyPhen2 [17], SIFT [18], PROVEAN [19], and Mutation-Taster [20] for the interpretation of sequence variants.

Complement factor H risk haplotype H3 (*CFH*-H3) was diagnosed in cases where three variants were detected (rs3753394, rs3753396, and rs1065489), and if only rs3753396 and rs1065489 were present, *CFH*-H8 was diagnosed. *CD46* risk haplotype ggaac (*CD46*ggaac) was diagnosed if five variants (rs2796267, rs2796268, rs1962149, rs859705, and rs7144) were present [21].

To further assess the genetic risk of end-stage renal disease (ESRD), we classified patients into genetic risk categories, according to a modified, previously published, algorithm [13]. We used 3 risk categories (low, medium, high) to assess the risk of ESRD according to detected variants and risk haplotypes. The genetic risk categories were as follows: 1 (no or low risk)—no variants, except for isolated variants in *CD46*, *DGKE*, or an isolated heterozygous *CD46*ggaac or heterozygous *CFH*-H3 risk haplotype; 2 (moderate risk)—isolated variants in any gene (*CFH*, *CFI*, *C3*, *CFB*, *THBD*, *CFHR* 1-5; except *CD46* or *DGKE*) or the homozygous *CFH*-H3 or homozygous *CD46*ggaac risk haplotype; and 3 (high risk)—variants in any gene together with either *CFH*-H3 or *CD46*ggaac risk haplotypes or a combination of variants and risk haplotypes [22].

### 2.4. Therapy of cTMA

Patients received either plasma exchange (PE) or plasma infusions (PI), eculizumab, or supportive therapy, based on clinical judgment and the availability of treatment methods. Plasma exchange was defined as a minimum of three sessions over five days and plasma infusions as a minimum of five infusions over five days [23]. When the C5-antibody eculizumab was used, it was administered according to the manufacturer’s prescribing information [5,24]. Plasma exchange has been used as a first line of therapy since it became available as a routine therapy in the mid-1980s. Since 2012, eculizumab has been available, and it is used in cases of failure or intolerance of PE for all patients with suspected or proven cTMA; a detailed description of the treatment approach can be found in Aigner et al. [1].

### 2.5. Statistical Analysis

Data are presented as count and frequency or as mean and standard deviation. For comparison of means, the Student’s t-test was used for normally distributed values and nonparametric tests were used for non-normally distributed data. Survival data were estimated by means of Kaplan–Meier plots and analyzed by performing log-rank tests. To adjust for possible confounders, we performed multivariate Cox regression models to determine the influence of sex on renal survival, while adjusting for other covariables (female patients and supportive treatment were used as reference variables). Cases that were neither on renal replacement therapy (RRT), according to the Austrian Dialysis and Transplant Registry (OEDTR), nor deceased with missing estimated glomerular filtration rate (eGFR) were defined as chronic kidney disease (CKD). Kidney transplantation was defined as another type of RRT. All women older than the age of 16 years at first disease manifestation were included for calculations concerning pregnancy-associated cTMA. We used MS Excel and IBM SPSS 24.0 for data management and analysis. A two-tailed *p*-value of <0.05 was considered statistically significant.

## 3. Results

### 3.1. Patients with cTMA Enrolled in the Vienna TMA Cohort

**Figure 1:**
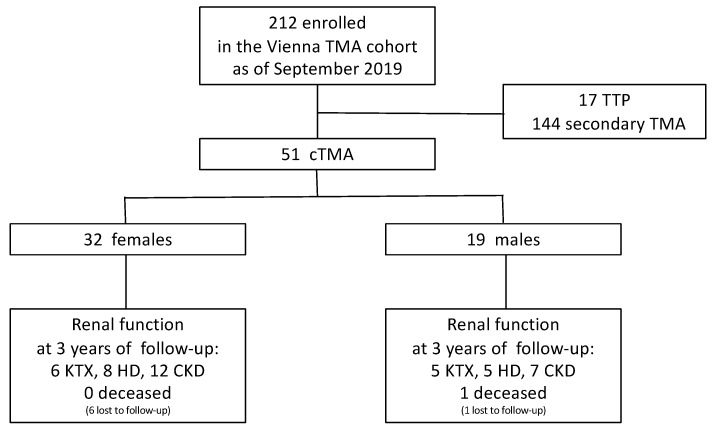
Patients enrolled in the Vienna thrombotic microangiopathy (TMA) cohort. Abbreviations: TMA, thrombotic microangiopathy; cTMA, complement-gene-variant-mediated thrombotic microangiopathy; TTP, thrombotic thrombocytopenic purpura; KTX, kidney transplantation; HD, hemodialysis; CKD, chronic kidney disease.

Between 1981 and September 2019, a total of 51 patients with a clinical diagnosis of cTMA were enrolled in the Vienna TMA cohort (Figure 1). The demographic and clinical details are indicated in Table 1. The median age at presentation was 28 years for both females and males. Sixty-three percent were female, and the majority of included patients were older than 18 years at the time of first disease manifestation (78%). Two patients had a family history of cTMA, and 19 patients (38.3%) had recurrent cTMA. In total, 10 female patients (31%) presented with cTMA during pregnancy or shortly after delivery.

### 3.2. Sex Differences in Renal Function During Presentation and Follow-Up of cTMA

The median age at presentation was 27.5 years for female patients, while the median age for male patients was 29 years (*p* = 0.93) (Figure S1, Table 2. The male-to-female ratio was 1:1.68 in the whole cohort, 1:1.85 in the adult population, and 1:1.2 in the patients <18 years of age at disease manifestation. In total, 10 female patients had their first disease episode during or shortly after pregnancy. Excluding patients with pregnancy-related cTMA manifestations, the adult male-to-female ratio was 1:1.16, which resembles the cohort of patients under the age of 18. The majority of patients of both sexes (29 females, 19 males) presented with acute kidney injury. Eighteen (56.3%) of 32 female patients and 12 (63.2%) of 19 male patients were dependent on RRT within 3 days of first presentation (Figure S2). Of the female patients, 15 received PE, 5 received PE followed by eculizumab, 1 received PI, and 11 patients were treated with supportive measures. Male patients were treated with PE in 5 cases, 2 were switched from PE to eculizumab, and 12 received supportive treatment (Table 3). Six female and three male patients recovered their kidney function and RRT could be terminated at the time of hospital discharge. Those six female patients were treated with PE in four cases and by PE followed by eculizumab in two cases. The three male patients were treated with PE, PE followed by eculizumab, and with supportive measures, respectively. One male patient died during the first cTMA episode and was the only patient who tested positive for anti-CFH autoantibodies. We could not detect a difference in treatment responses concerning the probability of ESRD after 1 and 3 years between male and female patients (hazard ratio (HR) 0.97 (95% confidence interval: 0.63–1.50); Table 4). No difference in the patients treated with eculizumab concerning the probability of ESRD after 1 and 3 years between male and female patients could be detected using the log-rank test-based regression (*p* = 0.82).

**Figure 2:**
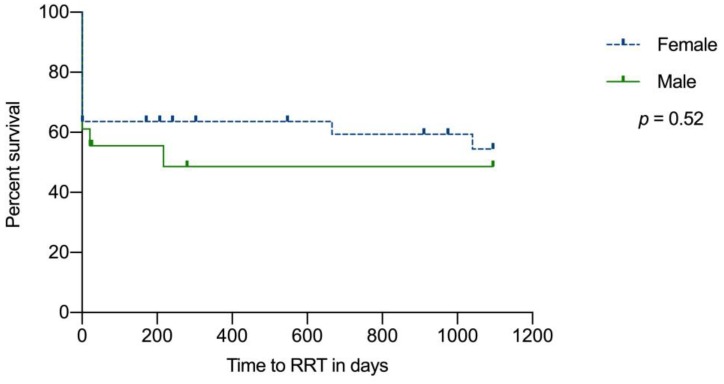
Renal survival of patients with cTMA enrolled in the Vienna TMA cohort according to sex. Abbreviations: cTMA, complement-gene-variant-mediated thrombotic microangiopathy; TMA, thrombotic microangiopathy; RRT, renal replacement therapy.

At hospital discharge, 11 female patients were dependent on RRT, and 14 and 15 female patients were dependent on RRT after one and three years of follow-up, respectively (Figure 2). In male patients, nine were on RRT at hospital discharge and one patient died during the first disease manifestation. After both 12 and 36 months of follow-up, 10 male patients were dialysis dependent. A multivariate Cox regression model showed a hazard ratio (HR) of 1.44 and, therefore, a trend toward an increased risk of ESRD for male patients. However, no statistically significant influence of sex, genetic risk category, or treatment method on the risk of ESRD after 12 and 36 months could be shown (Table 4). A total of five female patients and one male patient were lost to follow-up after three years.

The mean eGFR (Chronic Kidney Disease Epidemiology Collaboration, CKD-EPI) in female patients was 63.5 (± 36.7) one year after presentation and was 65.5 (± 48.7) mL/min per 1.73 m^2^ after three years. For female patients, the mean time to eGFR < 60 mL/min per 1.73 m^2^ was 453 days (95% CI: 557–649). In male patients, the mean eGFRs (CKD-EPI) one and three years after presentation were 56.4 (± 39.4) and 75.0 (± 34.7) mL/min per 1.73 m^2^, respectively. Male patients had a mean time to eGFR < 60 mL/min per 1.73 m^2^ of 243 days (95% CI: 25–461) (*p* = 0.08) (Figure S3).

After 12 months of follow-up, no patient had received a renal transplant; however, after 36 months, six female and five male patients had received a kidney graft (Figure S4).

We could not detect a statistically significant difference for renal outcomes between sexes (please refer to Table 2 and Table 4).

### 3.3. Sex Differences in Genetics

Genetic analysis of genes associated with cTMA were available in 50 patients (32 females, 18 males). Details of the genetic analysis of all patients are indicated in Table S1. Genetic analysis was not available in one male patient who tested positive for anti-CFH antibodies. Rare genetic variants were detected in 19 (59%) female and 8 (42%) male patients, respectively (*p* = 0.38) (Table 5).

In female patients, rare genetic variants in *CFH*, *CD46*, and *C3* were most frequently diagnosed (each *n* = 4), followed by variants in *CFHR5* (*n* = 2) and in *THBD*, *DKGE*, and *CFI* (each *n* = 1). Whereas in the male population, variants in *CFH* and *CFI* were most frequently diagnosed (each *n* = 3), followed by variants in *CD46* and *C3* (each *n* = 1), and no rare variants were detected in the remaining genes (Figure S5).

If variants were classified according to the ACMG, we detected eight variants of unknown significance (VUS), five likely pathogenic, and six pathogenic variants in our female patients, while we detected four VUS, one likely pathogenic, and three pathogenic variants in male patients (*p* = 0.73).

According to the genetic risk score, 14, 9, and 9 female patients were categorized as high, medium, and low genetic risk of progressing to ESRD, respectively, while 7 male patients were categorized as high risk, 4 as medium risk, and 7 as low risk (*p* = 0.81).

**Figure 3:**
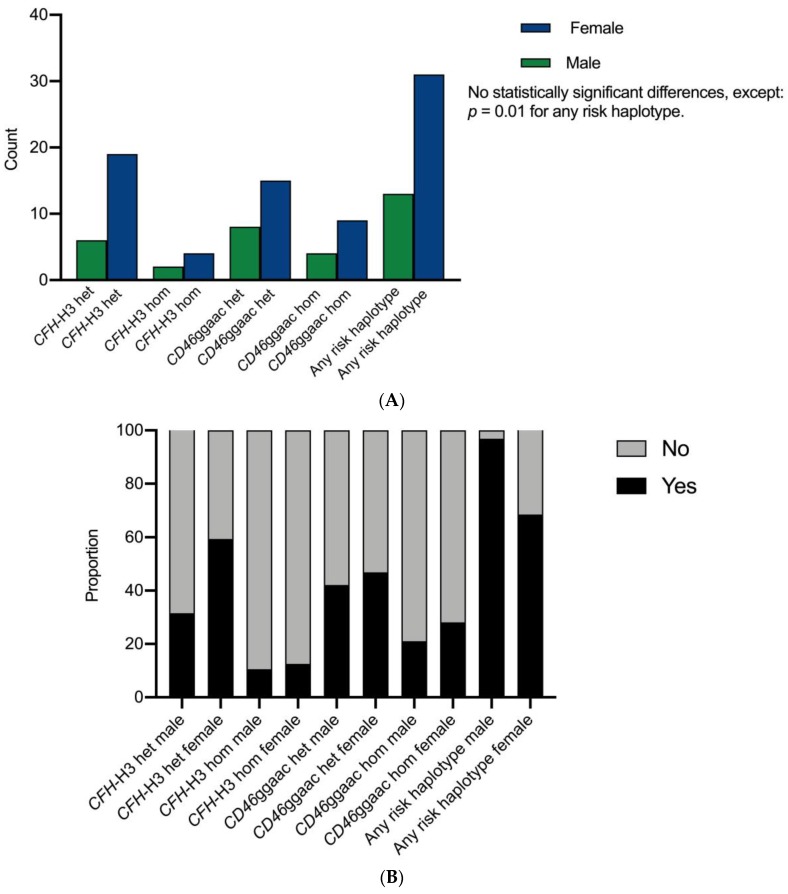
(**A**) count and (**B**) proportion of *CFH*-H3 and *CD46*ggaac risk haplotypes in patients diagnosed with cTMA according to sex. Abbreviations: het, heterozygous; hom, homozygous. *CFH*-H3, complement factor H risk haplotype H3; *CD46*ggaac, *CD46* risk haplotype ggaac.

Nineteen female patients tested positive for the heterozygous variant of the *CFH*-H3 risk haplotype and four for the homozygous variant; in contrast, six male patients tested positive for the heterozygous variant of *CFH*-H3 and two for the homozygous variant (*p* = 0.15). In addition, 15 females were heterozygous and 9 were homozygous for the *CD46*ggaac risk haplotype, while 8 and 4 male patients were heterozygous and homozygous for *CD46*ggaac, respectively (*p* = 0.8; Figure 3). Eighteen female and seven male patients tested positive for any genetic risk haplotype (*p* = 0.01).

Furthermore, patients were classified according to genetic risk for ESRD. In total, 14 female and 7 male patients were categorized as high risk, 9 female and 4 male patients as medium risk, and 9 female and 7 male patients as low risk (Figure S6).

Female patients classified as low risk had a mean renal survival of 598 days (95% CI: 248–948), whereas patients classified as medium risk had a mean renal survival of 973 days (95% CI: 655–1291) and high-risk patients had a mean renal survival of 584 days (95% CI: 309–860) (*p* = 0.91). In contrast, male patients classified as low risk had a mean renal survival of 629 days (95% CI: 230–1028), whereas patients classified as medium risk had a mean renal survival of 328 days (95% CI: 0–770) and high-risk patients had a mean renal survival of 469 days (95% CI: 68–871) (*p* = 0.60).

## 4. Discussion

Our study shows that the majority of cTMA patients enrolled in the Vienna TMA cohort were female. Clinical presentation and renal function did not differ between the sexes, but females more frequently presented with cTMA risk haplotypes in *CFH* and *CD46*.

### 4.1. Sex and Age at Presentation

Our study of 51 patients with cTMA, shows a female preponderance of 63%, mainly due to female patients affected by p-cTMA. Previous studies have shown that more adult female than male patients present with cTMA or secondary TMA [11,21,25,26,27,28]. In a large study of 273 patients with primary and secondary TMA, sex was indicated for 225 patients. The male-to-female ratio in these cases was 125:120 (1:1.04), and sex was not examined as a disease modifying factor [25]. In the study by Bresin et al., there were more males among patients with combined cTMA genetic variants [29]. Another large study of 214 pediatric and adult patients with cTMA from Fremeaux-Bacchi et al. showed a male-to-female ratio of 1:2.9 (32:93) in adults and of 1:0.9 (47:42) in children [26]. Of interest, in 18 out of 93 females enrolled in this study, the onset of cTMA was associated with pregnancy [26]. In another analysis from France of 110 patients with secondary TMA, the male-to-female ratio was 1:1.44 (45:65) [27]. An update of a French cTMA cohort recently showed females to predominate males with a male-to-female ratio of 1:1.78 (143:254) [28]. Similarly, Szarvas et al. described more female than male patients with a male-to-female ratio of 1:1.3 (13:17) in a cohort of cTMA patients with eastern European descent [21]. In one of the largest studies of 851 patients with cTMA, including 14% with secondary TMA, enrolled in the Global aHUS Registry, the male-to-female ratio at childhood presentation was 1.3:1 and 1:2 for adult presentation. Male patients with aHUS were younger (median age: 10.0 years) at initial presentation than female patients (median age: 25.6 years). Sex, however, showed no association with ESRD risk, and the effect of age on patient survival was not considered in this analysis [11]. Finally, Osborne and colleagues described 1231 cTMA/aHUS patients, enrolled from six reference centers, with at least one identified rare variant in complement-alternative-pathway-associated genes [9]. Among patients with known sex, 36% (440) were female and 29% (363) were male. However, when patients with *CFH* variants were removed from the analyses, the number of females (244) and males (225) was not significantly different, showing a trend toward more females for *CFH* variants. For example, in p-cTMA, most patients harbor *CFH* variants [7,8]. Summarizing the abovementioned studies, it appears that there is a female predominance among adult cTMA patients, which is likely related to the number of p-cTMA cases.

### 4.2. Renal Survival and cTMA Specific Therapy

The influence of biological sex on renal or patient survival in cTMA patients has not been analyzed in the majority of previously published studies. To our knowledge, only Schäfer et al. examined the differences in the ESRD-free survival probability of male and female patients and could not find a statistically significant difference [11]. In our cohort, we could not find a difference in renal outcomes at the time of discharge or after one and three years of follow-up. However, male patients had a shorter mean time to eGFR < 60 mL/min per 1.73 m^2^ (243 days; 95% CI: 45–461) than female patients (453 days; 95% CI: 557–649; *p* = 0.08). Furthermore, the HR for ESRD showed a trend toward a higher risk for male patients, which increased slightly after adjusting for genetic risk category, which could indicate a higher awareness for cTMA in female patients. Of course, a random effect cannot be ruled out. In addition, no significant difference in the number of kidney transplant recipients within three years of follow-up was detected.

No statistically significant difference between genders concerning treatment regimens could be detected. In the overall cohort, 21 patients were treated with PE as therapy for cTMA. Of those, 12 patients were initially dependent on RRT and 7 were still on RRT after 12 months. This shows a comparable success rate of plasma therapy compared to previously published data [29]. The large number of patients treated with supportive measures can be explained by the large timespan of disease presentation covered by this study (i.e., PE was not readily available in the 1980s). In 2012, eculizumab became available for the treatment of cTMA patients in Austria. The mean renal survival was longer after 2012 than before 2012; however, this difference was not statistically significant (*p* = 0.06). Of the seven patients that received eculizumab at first disease presentation during the reported time, two were male and five female. Of those, one male and one female patient were dependent on chronic RRT after 12 months. Furthermore, one patient recovered their renal function under therapy. No difference in treatment response could be detected between sexes.

### 4.3. Genetic Variants

Genetic variants in genes associated with complement function or regulation are well-known susceptibility factors for cTMA. Previous data have shown a trend toward a higher prevalence of rare variants in *CFH* (which tend to be the most deleterious considering renal and patient survival) in female patients. However, other analyses considering this topic are lacking. In our cohort, we found a similar prevalence of rare variants in female and male patients. Conversely, we found that female patients were more likely to harbor either a *CHF*-H3 or *CD46*ggaac risk haplotype than male patients (31 females, 13 males; *p* = 0.01). These at-risk haplotypes increased the risk for cTMA fourfold and threefold, respectively, in a large French cohort [26]. The clinical relevance of this finding needs to be validated in a larger cohort. As in one of our previous studies, we categorized patients according to genetic variants into three risk categories, taking all detected variants as well as risk haplotypes into account [13]. However, we did not find a difference in risk for ESRD between sexes and the three genetic risk groups.

### 4.4. Limitations

We are aware of several limitations that are inherently bound to a study of retrospective nature, and these include the large timespan of reviewed cases (i.e., specific therapies were not available in the 1980s) and the small case numbers compared to other multicenter analyses.

## 5. Conclusions

In conclusion, our data hints that pregnancy is a large contributing factor for why the majority of adult patients are female. In our cohort, female patients tended to have superior kidney survival compared to male patients. We did not find any differences in the prevalence of rare genetic variants between male and female patients; however, female patients are significantly more likely to harbor a *CFH*-H3 or *CD46*ggaac risk haplotype.

## Figures and Tables

**Table 1:** Patient characteristics and clinical presentation.

Characteristic	All Patients (*n* = 51)
Age at presentation, years	28 (19–39)
Presentation < 18 years	11 (21.6%)
Female	32 (62.7%)
**Race**	
Caucasian	45 (88.2%)
Black	3 (5.9%)
Asian	3 (5.9%)
Family history	2 (4%)
RRT at presentation	30 (58.8%)
Chronic RRT after hospital discharge (initial presentation)	21 (41.2%)
Death from any cause in the first 3 years	1 (2%)
ESRD after 1 year	23 (45%)
ESRD after 3 years	24 (47%) ^1^
PE/PI at presentation	28 (39.3%)
Eculizumab after PE/PI at presentation	7 (15.7%)
Anti-FH autoantibodies	1 (2%)
Patients with relapsing disease	19 (38.3%)
Kidney transplant recipients within 3 years	11 (21.5%)
Genetic variants (including risk haplotypes) *n*/*n* screened	48/50 (94%) ^2^
Rare variants, *n*/*n* screened	27/50 (54%)
*CFH*-H3, *n*/*n* screened	31/50 (62%)
Heterozygous	25 (50%)
Homozygous	6 (12%)
*CD46*ggaac, *n*/*n* screened	36/50 (72%)
Heterozygous	23 (46%)
Homozygous	13 (26%)

Abbreviations: RRT, renal replacement therapy; ESRD, end-stage renal disease; PE/PI, plasma exchange/plasma infusions; FH, factor H; *CFH*, complement factor H; *CFH*-H3, complement factor H risk haplotype H3; *CD46*ggaac, *CD46* risk haplotype ggaac. ^1^ No 3-year follow-up data for seven patients; ^2^ Individual genetic data given in supplementary material.

**Table 2:** Sex differences in renal function at presentation and during follow-up.

Characteristic	Female	Male	*p*
Patients	32 (63%)	19 (37%)	0.09
Patients presenting with p-cTMA	10 (32%)	n.a.	
Age at diagnosis, years (median)	27.5	29	0.96
**Kidney function at presentation**			
AKI	29 (91%)	14 (74%)	
Acute RRT	18 (56%)	12 (63%)	0.63
**Kidney function at 1-year follow-up**			0.28
Chronic kidney disease	19 (59%)	8 (42%)	
Dialysis	13 (41%)	10 (53%)	
Deceased	0	1 (5%)	
Kidney transplant	0	0	
**Kidney function at 3-year follow-up ^1^**			0.34
Chronic kidney disease	12 (38%)	7 (37%)	
Dialysis	8 (25%)	5 (26%)	
Deceased	0	1 (5%)	
Kidney transplant	6 (19%)	5 (26%)	

Abbreviations: p-cTMA, pregnancy-associated complement-gene-variant-mediated thrombotic microangiopathy; n.a., not applicable; AKI, acute kidney injury; RRT, renal replacement therapy. ^1^ Shorter follow-up than 36 months or lost to follow-up in six female patients and one male patient.

**Table 3:** Therapy of cTMA at presentation.

Characteristic	Female (*n* = 32)	Male (*n* = 19)	*p*
**Treatment at presentation**			0.15
PE/PI	16 (52%)	5 (26%)	
Eculizumab after PE	5 (16%)	2 (11%)	
Supportive	11 (34%)	12 (63%)	

Abbreviations: PE, plasma exchange; PI, plasma infusion.

**Table 4:** Predictors of ESRD after 1 (Model 1) and 3 (Model 2) years (multivariate Cox regression).

Variables	Crude HR	Adjusted HR	Confidence Interval	*p*
**Model 1**				
Sex	1.44	1.44	0.63–3.30	0.39
Genetic risk category		1.02	0.63–1.65	0.94
Treatment		0.97	0.63–1.50	0.89
**Model 2**				
Sex	1.254	1.33	0.59–3.0	0.49
Genetic risk category		1.07	0.67–1.72	0.79
Treatment		0.95	0.61–1.47	0.8

Abbreviations: ESRD, end-stage renal disease; HR, hazard ratio.

**Table 5:** Summary of genetic variants.

Characteristic	Female	Male	*p*
Patients	32 (63%)	19 (37%)	0.09
Rare genetic variant ^1^	19 (59%)	8 (42%)	0.38
**ACMG category**			0.93
Pathogenic	6 (19%)	3 (16%)	
Likely pathogenic	5 (16%)	1 (5%)	
Variant of unknown significance	8 (25%)	4 (21%)	
Genetic risk category			0.81
Low risk (Category 1)	9 (28%)	7 (37%)	
Medium risk (Category 2)	9 (28%)	4 (21%)	
High risk (Category 3)	14 (44%)	7 (37%)	
*CFH*-H3			0.15
Heterozygous	19 (59%)	6 (32%)	
Homozygous	4 (13%)	2 (11%)	
*CD46*ggaac			0.8
Heterozygous	15 (47%)	8 (42%)	
Homozygous	9 (28%)	4 (21%)	
*CFH*-**H3 and***CD46***ggaac**			0.24
Heterozygous and heterozygous	8 (25%)	4 (21%)	
Heterozygous and homozygous	6 (19%)	1 (5%)	
Homozygous and heterozygous	2 (6%)	2 (11%)	
Homozygous and homozygous	0	0	
*CFH*-H3 or *CD46*ggaac	31 (97%)	13 (68%)	0.01

Abbreviation: ACMG, American College of Medical Genetics and Genomics. ^1^ No data available for one male patient.

## Supplementary Materials

The following are available online at https://www.mdpi.com/2077-0383/9/4/964/s1: Figure S1: Age at presentation with complement-gene-variant-mediated TMA of patients enrolled in the Vienna TMA cohort according to sex; Figure S2: Count of patients on renal replacement therapy at presentation according to sex; Figure S3: Patients with complement-gene-variant-mediated TMA enrolled in the Vienna TMA cohort with an eGFR greater than 60 mL/min per 1.73 m2 according to sex; Figure S4: (A) Count and (B) proportion of kidney transplants among female and male patients with cTMA enrolled in the Vienna TMA cohort; Figure S5: Count of patients enrolled in the Vienna TMA cohort with cTMA due to rare variants in (A) CFH and (B) all relevant genes according to sex; Figure S6: Time to renal replacement therapy in days for each genetic risk category (low, medium, high) for a total follow-up of three years; Table S1: Summary of genetic variants of 50 patients with cTMA.
