# Supplementary material for: Sex Differences in Clinical Presentation and Outcomes among Patients with Complement-Gene-Variant-Mediated Thrombotic Microangiopathy
Source: J Clin Med. 2020 Mar 31;9(4):964. doi: 10.3390/jcm9040964 (PMC7230736; doi:10.3390/jcm9040964)
Supplement: Supplementary file 1 [file jcm-09-00964-s001.pdf]

Table 1. Summary of genetic variants of 50 patients with cTMA.

Patient	Sex	Gene	Alteration in Protein	Domain	Minor Allele Frequency	Functional Effect	Risk Haplotype	Copy Number of *CFH CFHR-1,2,3,5*	Genetic Risk Category (1, 2, 3)
1	M	none	-	-	-	-	none	no alteration	1
2	F	none	-	-	-	-	*CD46ggaac*, het	no alteration	1
3	F	none	-	-	-	-	*CFH*-H3, het	no alteration	1
4	F	none	-	-	-	-	*CFH*-H3, het; *CD46*ggaac, het	no alteration	1
5	F	*THBD*	p.E560Q het (LPV)		0.01–0.02%	not characterized	*CFH*-H3, het	no alteration	3
6	M	none	-	-	-	-	*CD46*ggaac, het	het del*CFHR1,3*	1
7	F	*CFHR5*	p.G228A het (VUS)		0.01%	not characterized	*CFH*-H3, hom	no alteration	3
8	F	none	-	-	-	-	*CFH*-H3, hom	no alteration	1
9	M	none	-	-	-	-	none	hom del*CFHR1,3*	1
10	M	*CFI*	p.T203I het (VUS)	SRCR	0.08%	not characterized	none	no alteration	3
11	F	*CFH*	p.N1050Y * het (VUS) [1]	SCR20	0.01%	not characterized	*CD46*ggaac het	het del*CFHR1,3*	3
12	F	none	-	-	-	-	*CFH*-H3, het; *CD46*ggaac, hom	no alteration	1
13	F	*CD46*	p.A353V het (P)	TM	0.29–1.73%	deficient cell surface control of AP [2]	*CD46*ggaac, hom	no alteration	2
14	M	none	-	-	-	-	*CD46*ggaac, hom	het del*CFHR1,3*	2
15	M	none	-	-	-	-	*CD46*ggaac, het	het del*CFHR1,3*	1
16	F	C3	p.D61N het (LPV)	MG1	0%	not characterized	*CFH*-H3, het; *CD46*ggaac, hom	no alteration	3
17	F	*CFH*	p.C1032 * het (P)	SCR20	0%	stop codon	*CD46*ggaac, hom; *CFH*-H8, het	het del*CFHR1,3*	3
18	F	*DGKE*	c.1284 + 151A > G (LPV)	n.a.	0.10%	n.a.	*CFH*-H3, hom	no alteration	2
19	F	none	-	-	-	-	*CFH*-H3, het	no alteration	1

20	F	*CD46*	p.E142Q het (VUS)	SCR2	0%	no effect on *CD46* expression or cofactor function [3]	*CFH*-H3, het	het del*CFHR1,3*	2
21	F	none	-	-	-	-	-	het del*CFHR1,3*	1
22	M	*CFH*	p.S1191L het *(P)	SCR20	0%	deficient cell surface	*CFH*-H3, het		3
		CFH	p.V1197A het *(P)	SCR20	0%	control of AP [3–5]	*CD46*ggaac, het	conversion *CFH, CFHR-1*	
23	M	*CFI*	p.G263V het (VUS)	LDLR2	0%	not characterized	*CFH*-H3, het; CD46ggaac, het	no alteration	3
24	F	*CFHR5*	c.479_480insA het (VUS)	SCR3	0%	not characterized	*CD46*ggaac, het	no alteration	3
25	M	none	-	-	-	-	*CFH*-H3, het	no alteration	1
26	F	*CFH*	p.N516K het (LPV)	SCR9	0.02–0.04%	not characterized	*CD46*ggaac, hom	no alteration	3
							CFH c.331C > T, het; *CFH*-H8, hom		
27	M	*CFH*	p.R1215Q het (P)	SCR20	0%	reduced binding to effectors [6]	*CFH*-H3, het	no alteration	3
28	F	none	-	-	-	-	*CFH*-H3, het; *CD46*ggaac, hom	no alteration	2
29	F	*CD46*	p.A353V hom (P)	TM	0.29–1.73%	deficient cell surface control of AP [2]	*CFH*-H3, het; *CD46*ggaac, hom	no alteration	2
30	F	*C3*	p.K104E het (VUS)	MG1	0%	not characterized	*CFH*-H3, het; *CD46*ggaac, het	no alteration	3
31	F	*CFI*	p.I416L het (P)	serine protease	0.01–1.29%	quantitative FI deficiency [7]	*CD46*ggaac, het	het del*CFHR1,3*	3
32	F	*C3*	p.V1296A het (VUS)	CUBb	0%	not characterized	*CFH*-H3, het; *CD46*ggaac, het	het del*CFHR1,3*	3
33	F	none	-	-	-	-	*CFH*-H3, het; *CD46*ggaac, hom	n.a.	2
34	F	none	-	-	-	-	*CD46*ggaac, het	no alteration	1
35	M	*C3*	p.I1157T het (VUS)	TED	0%	altered binding to FH and MCP [8,9]	*CD46*ggaac, hom	het del*CFHR1*	3
36	F	*C3*	p.I1157T het (VUS)	TED	0%	altered binding to FH and MCP	*CD46*ggaac, hom	het del*CFHR1*	3

37	M	none	-	-	-	-	*CFH* H3, het; *CD46*ggaac, hom	no alteration	2
38	F	none	-	-	-	-	*CFH* H3, het; *CD46*ggaac, hom	no alteration	2
39	M	none	-	-	-	-	none	no alteration	1
40	F	none	-	-	-	-	*CFH* H3, het	no alteration	1
41	F	none	-	-	-	-	*CFH* H3, hom; *CD46*ggaac, het	no alteration	2
42	F	*CFI*	p.G342E het (LP)	SP	0%	not characterized	*CFH* H3, het; *CD46*ggaac, het	no alteration	3
		CD46	p.D257V[a]fs 418 (LP)	SCR4	0%	not characterized			
43	M	none	-	-	-	-	*CFH* H3, het; *CD46*ggaac, hom	n.a.	2
44	F	*CD46*	p.E234K (VUS)	SCR4	0%	not characterized	*CFH* H3, het; *CD46*ggaac, het	n.a.	2
45	M	*CD46*	p.A353V het (P)	TM	0.29–1.73%	deficient cell surface	*CFH* H3, hom	het del *CFHR1,3*	2
						control of AP [2]	*CD46*ggaac, het		
46	M	*CFI*	p.R406H het (VUS)	SP	0%	not characterized	none	no alteration	3
47	F	*CFH*	p.S1191L het (P)	SCR20	0%	reduced promotion of hemolysis of C3b-coated erythrocytes [5]	*CFH* H3, het; *CD46*ggaac, het	no alteration	3
48	F	*CFH*	p.D748Nfs[a] 10 het (P)	SCR13	0%	fs causing, damaging	*CFH* H3, het	no alteration	3
		CD46	p.A353V het (P)	TM	0.29–1.73%	deficient cell surface	*CD46*ggaac, het		
					control of AP				
49	M	none	-	-	-	-	*CFH* H3, het; *CD46*ggaac, het	n.a.	1
50	M	n.a.	-	-	-	-	-	-	n.a.
51	M	*CFH*	p.C1048Y het (LPV)	SCR20	0%	not characterized	*CFH* H3, hom; *CD46*ggaac, het	no alteration	3

Abbreviations: cTMA, complement-gene variant mediated thrombotic microangiopathy; M, male; F, female; *CFH*, complement factor H; *CFHR-1,2,3,5*, complement-factor-H-related protein 1,2,3,5; het, heterozygous; del, deletion; hom, homozygous; *CFI*, complement factor I; *THBD*, thrombomodulin; n.a., not available; TM, transmembrane; fs, frameshift; MG1, macroglobulin 1; AP, alternative complement pathway; SCR, short consensus repeat; SRCR, scavenger receptor cysteine-rich family; LPV, likely pathogenic variation; VUS, variation of unknown significance; P, pathogenic; LDLR2, LDL receptor 2; FI, Factor I; CUBb; CUB domain b; TED, Thioester-containing domain;

SP, serine protease. * These alterations arise by a gene conversion between *CFH* and *CFHR-1* and are regarded as one mutation [4]. Genetic risk categories: 1 (no or low risk): no variants, except for isolated variants in *CD46*, *DGKE*, or an isolated het. *CD46*ggaac or het. *CFH*-H3 risk haplotype; 2 (moderate risk): isolated variants in any protein (*CFH*, *CFI*, *C3*, *CFB*, *THBD*, *CFHR 1–5*; except *CD46* or *DGKE*) or the hom. *CFH*-H3 or hom. C*D46*ggaac risk haplotype; 3 (high risk): variants in any protein together with either *CFH*-H3 or *CD46*ggaac risk haplotypes or a combination of variants and risk haplotypes.

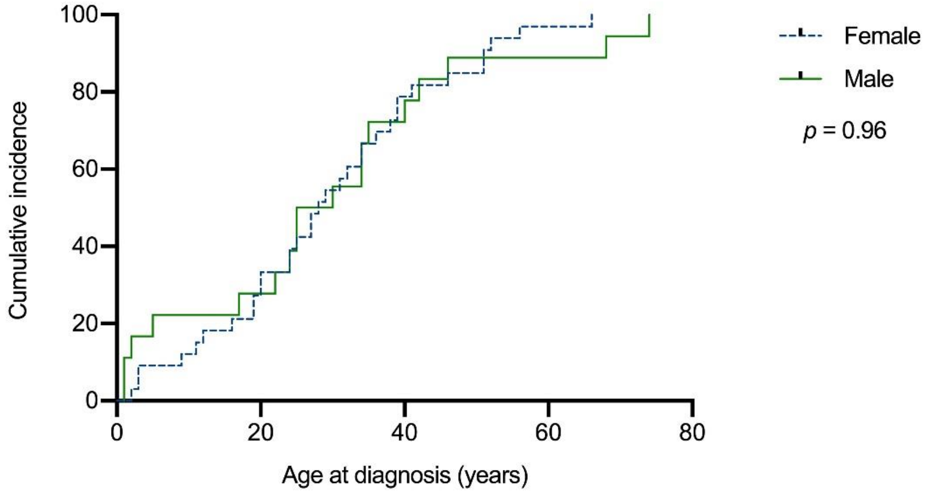

Figure S1. Age at presentation with complement-gene-variant-mediated TMA of patients enrolled in the Vienna TMA cohort according to sex. Abbreviation: TMA, thrombotic microangiopathy.

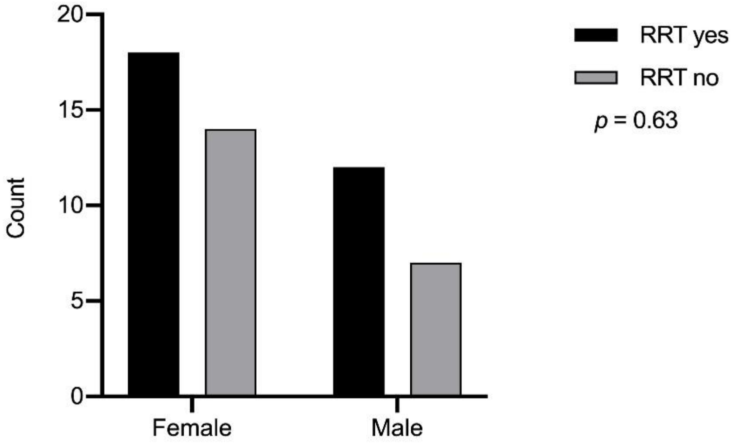

Figure S2. Count of patients on renal replacement therapy at presentation according to sex. Abbreviation: RRT, renal replacement therapy.

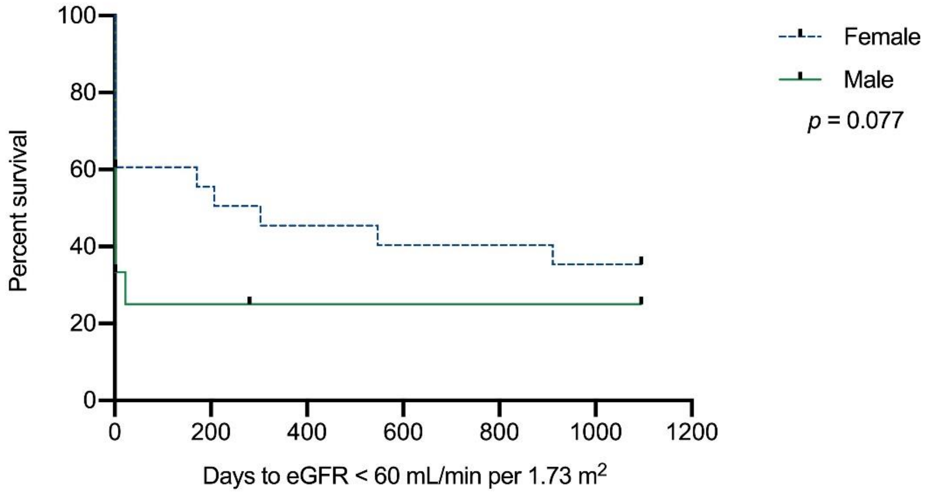

Figure S3. Patients with complement-gene-variant-mediated TMA enrolled in the Vienna TMA cohort with an eGFR greater than 60 mL/min per 1.73 m^2 according to sex. Abbreviations: TMA, thrombotic microangiopathy; eGFR, estimated glomerular filtration rate.

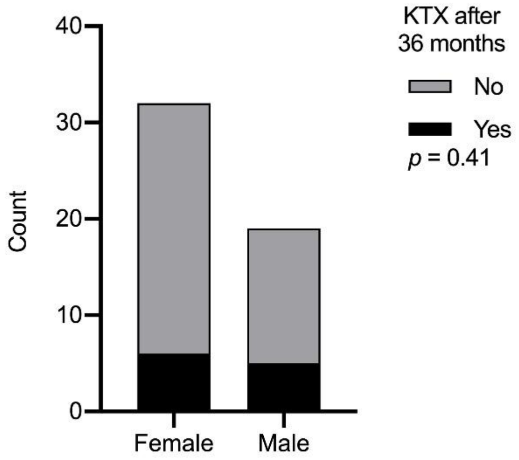

(**A**)

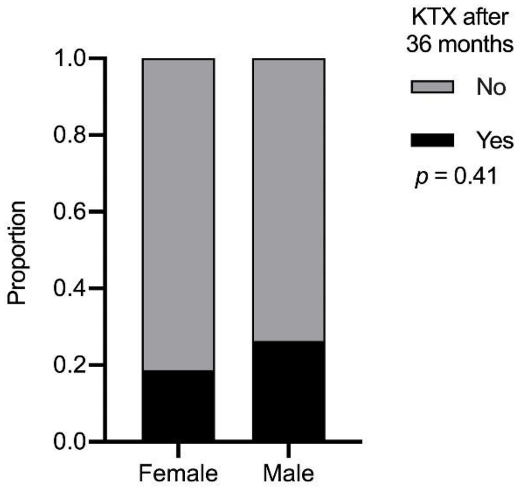

(**B**)

Figure S4. (**A**) Count and (**B**) proportion of kidney transplants among female and male patients with cTMA enrolled in the Vienna TMA cohort. Abbreviations: cTMA, complement-gene-variant-mediated thrombotic microangiopathy; KTX, kidney transplantation; TMA, thrombotic microangiopathy.

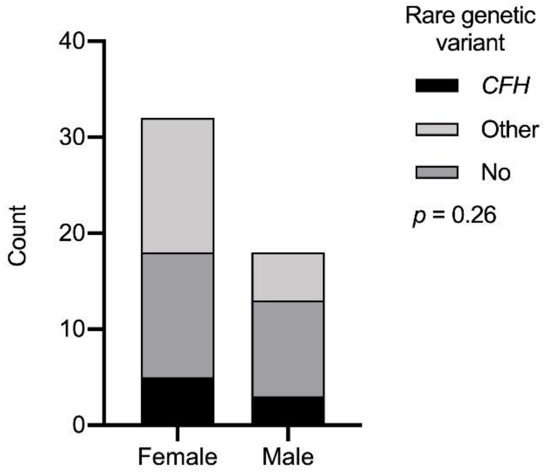

(**A**)

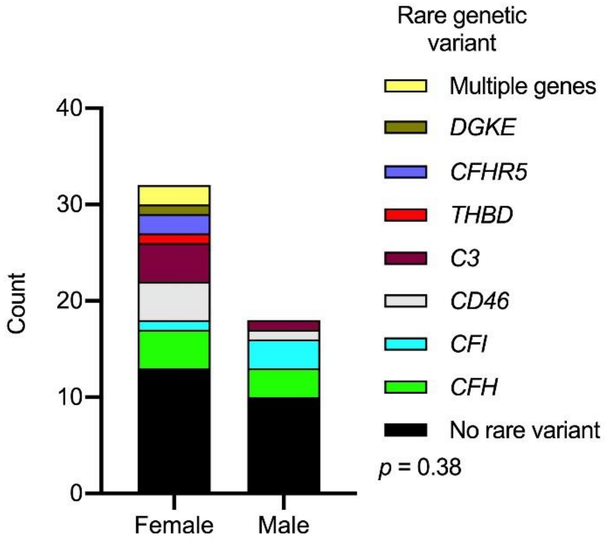

(**B**)

Figure S5. Count of patients enrolled in the Vienna TMA cohort with cTMA due to rare variants in (**A**) *CFH* and (**B**) all relevant genes according to sex. Abbreviation: cTMA, complement-gene-variant-mediated thrombotic microangiopathy; *CFH*, Complement factor H; *DGKE*, Diacylglycerolkinase epsilon; *CFHR5*, Complement factor H related protein 5; *THBD*, Thrombomodulin; *CFI*, Complement factor I.

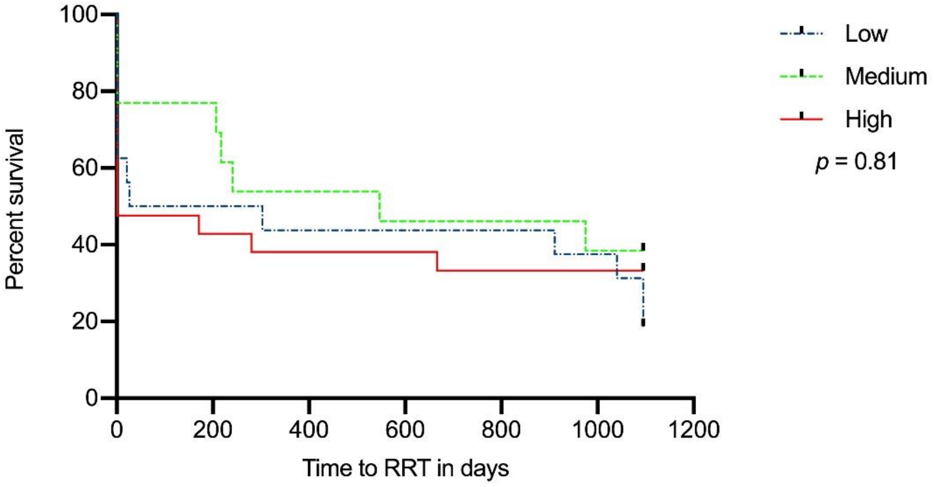

Figure S6. Time to renal replacement therapy in days for each genetic risk category (low, medium, high) for a total follow-up of three years. Abbreviation: RRT, renal replacement therapy.

References

1. Richards, A.; Buddles, M.R.; Donne, R.L.; Kaplan, B.S.; Kirk, E.; Venning, M.C.; Tielemans, C.L.; Goodship, J.A.; Goodship, T.H. Factor H mutations in hemolytic uremic syndrome cluster in exons 18-20, a domain important for host cell recognition. *Am. J. Hum. Genet.* **2001**, *68*, 485-490, doi:10.1086/318203.
2. Bresin, E.; Rurali, E.; Caprioli, J.; Sanchez-Corral, P.; Fremeaux-Bacchi, V.; Rodriguez de Cordoba, S.; Pinto, S.; Goodship, T.H.; Alberti, M.; Ribes, D., et al. Combined complement gene mutations in atypical hemolytic uremic syndrome influence clinical phenotype. *J. Am. Soc. Nephrol.* **2013**, *24*, 475-486, doi:10.1681/ASN.2012090884.
3. Mohlin, F.C.; Nilsson, S.C.; Levart, T.K.; Golubovic, E.; Rusai, K.; Müller-Sacherer, T.; Arbeiter, K.; Pallinger, E.; Szarvas, N.; Csuka, D., et al. Functional characterization of two novel non-synonymous alterations in CD46 and a Q950H change in factor H found in atypical hemolytic uremic syndrome patients. *Mol. Immunol.* **2015**, *65*, 367-376, doi:10.1016/j.molimm.2015.02.013.
4. Heinen, S.; Sanchez-Corral, P.; Jackson, M.S.; Strain, L.; Goodship, J.A.; Kemp, E.J.; Skerka, C.; Jokiranta, T.S.; Meyers, K.; Wagner, E., et al. De novo gene conversion in the RCA gene cluster (1q32) causes mutations in complement factor H associated with atypical hemolytic uremic syndrome. *Hum. Mutat.* **2006**, *27*, 292-293, doi:10.1002/humu.9408.
5. Herbert, A.P.; Kavanagh, D.; Johansson, C.; Morgan, H.P.; Blaum, B.S.; Hannan, J.P.; Barlow, P.N.; Uhrin, D. Structural and functional characterization of the product of disease-related factor H gene conversion. *Biochemistry* **2012**, *51*, 1874-1884, doi:10.1021/bi201689j.
6. Manuelian, T.; Hellwage, J.; Meri, S.; Caprioli, J.; Noris, M.; Heinen, S.; Jozsi, M.; Neumann, H.P.; Remuzzi, G.; Zipfel, P.F. Mutations in factor H reduce binding affinity to C3b and heparin and surface attachment to endothelial cells in hemolytic uremic syndrome. *J. Clin. Invest.* **2003**, *111*, 1181-1190, doi:10.1172/JCI16651.
7. Bienaime, F.; Dragon-Durey, M.A.; Regnier, C.H.; Nilsson, S.C.; Kwan, W.H.; Blouin, J.; Jablonski, M.; Renault, N.; Rameix-Welti, M.A.; Loirat, C., et al. Mutations in components of complement influence the outcome of Factor I-associated atypical hemolytic uremic syndrome. *Kidney Int.* **2010**, *77*, 339-349, doi:10.1038/ki.2009.472.
8. Martinez-Barricarte, R.; Heurich, M.; Lopez-Perrote, A.; Tortajada, A.; Pinto, S.; Lopez-Trascasa, M.; Sanchez-Corral, P.; Morgan, B.P.; Llorca, O.; Harris, C.L., et al. The molecular and structural bases for the association of complement C3 mutations with atypical hemolytic uremic syndrome. *Mol. Immunol.* **2015**, *66*, 263-273, doi:10.1016/j.molimm.2015.03.248.
9. Schramm, E.C.; Roumenina, L.T.; Rybkine, T.; Chauvet, S.; Vieira-Martins, P.; Hue, C.; Maga, T.; Valoti, E.; Wilson, V.; Jokiranta, S., et al. Mapping interactions between complement C3 and regulators using mutations in atypical hemolytic uremic syndrome. *Blood* **2015**, *125*, 2359-2369, doi:10.1182/blood-2014-10-609073.
